# Comparative Analysis of Root Traits and the Associated QTLs for Maize Seedlings Grown in Paper Roll, Hydroponics and Vermiculite Culture System

**DOI:** 10.3389/fpls.2017.00436

**Published:** 2017-03-30

**Authors:** Zhigang Liu, Kun Gao, Shengchen Shan, Riling Gu, Zhangkui Wang, Eric J. Craft, Guohua Mi, Lixing Yuan, Fanjun Chen

**Affiliations:** ^1^Key Lab of Plant-Soil Interaction, MOE, Center for Resources, Environment and Food Security, College Resources and Environmental Sciences, China Agricultural UniversityBeijing, China; ^2^Robert Holley Center for Agriculture and Health, USDA-ARSIthaca, NY, USA

**Keywords:** maize root, QTL, paper roll, hydroponics, vermiculite

## Abstract

Root system architecture (RSA) plays an important role in the acquisition of both nitrogen (N) and phosphorus (P) from the environment. Currently RSA is rarely considered as criteria for selection to improve nutrient uptake efficiency in crop breeding. Under field conditions roots can be greatly influenced by uncontrolled environment factors. Therefore, it is necessary to develop fast selection methods for evaluating root traits of young seedlings in the lab which can then be related to high nutrient efficiency of adult plants in the field. Here, a maize recombination inbred line (RILs) population was used to compare the genetic relationship between RSA and nitrogen and phosphorous efficiency traits. The phenotypes of eight RSA-related traits were evaluated in young seedlings using three different growth systems (i.e., paper roll, hydroponics and vermiculite), and then subjected to correlation analysis with N efficiency and P efficiency related traits measured under field conditions. Quantitative trait loci (QTL) of RSA were determined and QTL co-localizations across different growth systems were further analyzed. Phenotypic associations were observed for most of RSA traits among all three culture systems. RSA-related traits in hydroponics and vermiculite weakly correlated with Nitrogen (NupE) uptake efficiency (*r* = 0.17–0.31) and Phosphorus (PupE) uptake efficiency (*r* = 0.22–0.34). This correlation was not found in the paper roll growth system. A total of 14 QTLs for RSA were identified in paper rolls, 18 in hydroponics, and 14 in vermiculite. Co-localization of QTLs for RSA traits were identified in six chromosome regions of bin 1.04/1.05, 1.06, 2.04/2.05, 3.04, 4.05, and 5.04/5.05. The results suggest the problem of using the phenotype from one growth system to predict those in another growth system. Assessing RSA traits at the seedling stage using either hydroponics or a vermiculite system appears better suited than the paper roll system as an important index to accelerate the selection of high N and P efficient genotypes for maize breeding programs.

## Introduction

Roots play a significant role in the acquisition of water and mineral nutrients that are essential for plant survival in nature and yield production in agriculture (White and Brown, 2010). In maize, root system architecture (RSA) is a key determinant of water and nutrient uptake efficiency and is described as the organization of the primary root and root- and stem-derived branches (Hochholdinger and Zimmermann, 2008). The hypothetical ideotype RSA of maize for efficient nutrient acquisition which has been proposed, is that optimal numbers of and steeper angles of crown roots could modulate rooting depth and subsequently enhance water and nitrogen (N) acquisition (Mi et al., 2010; Lynch, 2011, 2013; Trachsel et al., 2013; Saengwilai et al., 2014). Furthermore, Hammer et al. (2009) demonstrated that changes in RSA had a direct effect on the increase of maize biomass yield production. Postma et al. (2014) also suggested that increasing lateral root branching density resulted in greater phosphorus (P) uptake. In addition, the phosphorus starvation-tolerance 1(*OsPSTOL1*) gene in rice, was found to enhance root length and surface area at the seedling stage. Lines overexpressing *OsPSTOL1* showed an increase of P uptake and approximately a 30% grain yield increase under low-P conditions (Gamuyao et al., 2012). Thus, it is promising to manipulate RSA toward a distribution of roots in the soil that optimizes nutrient uptake. This has become the subject of considerable interest in agriculture especially ensuring global food security in challenging environments (Lynch, 1995; de Dorlodot et al., 2007).

A thorough understanding of the genetic basis of RSA is the first key step in altering RSA toward better nutrient uptake. QTL mapping has been a major approach in investigating the genetic basis of maize root systems, as root traits are genetically controlled by a number of small-effect loci (de Dorlodot et al., 2007; Cai et al., 2012). Significant variation in RSA has been known to exist among maize genotypes, which provides an abundance of genetic material for QTL mapping (Jenison et al., 1981; Landi et al., 1998; Tuberosa et al., 2003; Chun et al., 2005; Kumar et al., 2012). After the first QTL study of maize RSA was conducted by Lebreton et al. (1995), numerous other work has been carried out under varying growth conditions, at different growth stages, and using different mapping populations (Hund et al., 2011). However, the localization of these QTL was inconsistent among the different studies. More QTL analyses for RSA was required to detect more loci and to ultimately identify consistent QTL for any future map-based cloning and marker associated selection.

Recent progress in DNA sequencing makes the acquisition of genotypic data an inexpensive, high-throughput procedure. However, phenotyping traits are the current bottleneck for RSA QTL analysis. Hence, a rapid and low-cost phenotyping method for RSA is urgently needed to fully capitalize on any potential genomic tools (Montes et al., 2007). RSA is complicated to be observed, quantified and interpreted directly in soil (Lynch, 1995; Trachsel et al., 2011). Since roots are a complex and dynamic entity growing in a heterogeneous and opaque soil they are greatly influenced by uncontrollable environmental factors. In addition, roots can be damaged when extracted from soil. Recently, several new methods have been developed for better evaluation of RSA in a field, such as “Shovelomics.” Some constraints still remain including, a long data acquisition time, the high cost of non-invasive imaging, and the limitation of detailed data on RSA from “Shovelomics” (de Dorlodot et al., 2007; Trachsel et al., 2011; De Smet et al., 2012).

As an alternative to field root studies, three indoor culture systems (paper roll, hydroponics and vermiculite) have been developed to allow for a rapid, low-cost, and non-destructive analysis of RSA at an early growth stage. Significant correlations between RSA and nutrient uptake efficiency were observed within the different culture systems. In the sand and vermiculite mixed system, maize lateral root length and axial root length had significant positive correlations with NupE in both high and low N treatment. Additional significant positive correlations were found between axial root length and PupE in high and low P treatment (DoVale et al., 2013). In the hydroponics system, seminal root number (SRN) showed significant correlations with NupE in a N deficient condition (Li et al., 2015). Under P deficiency, RSA-related traits such as SRN showed significant correlations with PupE (Gu et al., 2016). Although some of the correlations between RSA and nutrient uptake efficiency have been reported, the systematic comparisons between RSA and N/P uptake efficiency in different culture system are still scarce. Within the different culture systems, some RSA QTL's have been identified for the purpose of molecular breeding. Tuberosa et al. (2002b) demonstrated the feasibility of using a hydroponic growth system for maize seedlings to identify QTL regions controlling root traits. Hund et al. (2004) grew maize in a sand-vermiculite substrate to detect QTLs controlling both root and shoot growth. Zhu et al. (2005a,b, 2006) used paper roll cultures to identify QTLs associated with root and root hair related traits. Large differences existed among the three indoor culture systems, especially in terms of root stretching resistance, and the resource supply and distribution within the substrate. Given the observation of a strong root-environment interaction, it would be expected that the culture system will have an impact on RSA phenotype variation and QTL mapping. However, until now little was known about the comparison of RSA performance and the corresponding QTL among different culture systems.

In this study, we conducted a comparative phenotypic analysis and QTL identification in a maize recombinant inbred line (RIL) population grown in hydroponics, vermiculite and paper roll systems. The objective of this study was to (1) compare the phenotypic variation of root traits in the three culture systems; (2) investigate the phenotypic association between RSA traits and N efficiency- and P efficiency-related traits grown in field; (3) perform QTL mapping and further determine the QTL clusters for RSA traits from the different culture systems.

## Materials and methods

### Plant materials

A RIL population consisting of 218 F_8_ lines was used in this study. The population was derived from a cross between two inbred lines, Ye478 and Wu312. Previous studies showed that the root system of Ye478, as indicated by root biomass and total root length, was larger than that of Wu312 under both controlled and field conditions (Tian et al., 2006; Cai et al., 2012). A total of 184 simple sequence repeat (SSR) markers were used to construct a genetic linkage map which covered 2,064.9 cM with an average interval of 11.2 cM (Liu et al., 2011; Gu et al., 2016).

### Experiments in three culture systems

The RILs and their parents were grown in both hydroponics and paper rolls in a chamber with controlled climate conditions. The growth conditions were set with a photoperiod of 14/10 h (light/darkness), 28°/22°C (day/night) temperature, a photosynthetic photon-flux density of ~350 μmol m^−2^ s^−1^ at canopy height, and a relative humidity maintained at ~60%. Two independent experiments in hydroponics (H1 and H2) were conducted with six replications of each line. After sterilization with 2% (v/v) NaClO and soaked in saturated CaSO_4_, seeds were germinated for 2 days (d) in the dark on moist filter paper, and then wrapped in a paper roll similar to a cigar roll. The rolls were placed in buckets filled with distilled water, and cultivated in the dark for 2 day before being exposed to light. Uniform seedlings with two visible leaves were selected and transferred to plastic tanks containing 40 liters (L) of nutrient solution. Each tank contained 96 plants. Maize seedlings were harvested 12 and 13 days after germination in H1 and H2, respectively. Nutrients in the solution were (in mmol L^−1^): Ca(NO_3_)_2_·4H_2_O 2, K_2_SO_4_ 0.75, MgSO_4_·7H_2_O 0.65, KCl 0.1, KH_2_PO_4_ 0.25, MnSO_4_·H_2_O 1 × 10^−3^, ZnSO_4_·7H_2_O 1 × 10^−3^, CuSO_4_·5H_2_O 1 × 10^−4^, (NH_4_)_6_Mo_7_O_24_·4H_2_O 5 × 10^−6^, H_3_BO_3_ 1 × 10^−3^, Fe-EDTA 0.1. The pH of the solution was adjusted to 6.0 by using 1 M NaOH and HCl. The solution was aerated continuously and renewed every 3 days. Any positional effect within the growth chambers was minimized by rotating the tanks every second day. Two independent experiments in paper rolls (P1 and P2) were conducted with 8 and 15 replications of each line, respectively. The procedure of seed germination was the same as that described in hydroponics. However, the seedlings were continuously wrapped in the roll until harvested, which was 6 and 10 days after germination in P1 and P2, respectively.

The vermiculite culture experiment was conducted in a greenhouse with 17 replications of each line. Following the same germination procedure, uniform germinated seeds were selected and transplanted into plastic bucket containing vermiculite mixed with the same nutrient solution formulation used in the hydroponics study. Seedlings were harvested 10 days after germination.

### Root- and nutrient efficiency-related phenotype evaluation

All root traits investigated and their measurement methods are listed in Table 1. In brief, maize primary root, seminal root and crown roots (Hochholdinger and Zimmermann, 2008), were first measured with a ruler and indicated as PRL, SRL, and CRL, respectively. The number of seminal roots and crown roots were then counted and indicated as SRN and CRN, respectively. After measured the roots were scanned; and a total root length was determined using WinRHIZO Pro 2004b software (Regent Instruments, Canada). Lateral root lengths (LRL) were determined by subtracting the PRL, SRL, and CRL from the total root length. The number of lateral roots (>0.5 mm length) connected to the primary root was counted by eye. The length of the lateral root branching zone on the primary root was measured by a ruler. Lateral root density of the primary root (LRD_PR_) was calculated using the ratio of the number of lateral roots and the length of branching zone on primary root. The root dry weight (RDW) was measured after oven-drying at 65°C.

**Table 1:** **Summary of the investigated traits and the corresponding measurement methods**.

**Trait**	**Abbreviations**	**Units**	**Trait measurement**	**Data available**
**RSA-RELATED TRAITS IN THREE CULTURE SYSTEMS**				
Root dry weight	RDW	mg plant^−1^	Weighed after dried	In this study
The length of primary roots	PRL	cm	Measured with a ruler	
The length of seminal roots	SRL	cm	Measured with a ruler	
The number of seminal roots	SRN	number	Counted manually	
The length of crown roots	CRL	cm	Measured with a ruler	
The number of crown roots	CRN	number	Counted manually	
The length of lateral roots	LRL	cm	Scanned using image analysis software	
The lateral root density of primary root	LRD_PR_	cm^−1^	Number/length of primary roots	
**N EFFICIENCY RELATED TRAITS IN THE FIELD**				
N use efficiency	NUE	g/g	Grain yield/total amount of N supply	Li et al., 2015
N uptake efficiency	NupE	g/g	Total N uptake/total amount of N supply	
N utilization efficiency	NutE	g/g	Grain yield/total N content	
**P EFFICIENCY RELATED TRAITS IN THE FIELD**				
P use efficiency	PUE	kg/kg	Grain yield/total amount of P available in soil	Gu et al., 2016
P uptake efficiency	PupE	kg/kg	Total P uptake/total amount of P available in soil	
P utilization efficiency	PutE	kg/kg	Grain yield/Total P uptake	

Root phenotypic data on NUE- and PUE-related traits evaluated in the field were available from Li et al. (2015) and Gu et al. (2016), respectively (Table 1). The RSA traits of N use efficiency (NUE), N uptake efficiency (NupE) and N utilization efficiency (NutE) were investigated at both high- and low-N conditions (HN and LN) across four different environments (Li et al., 2015). The RSA traits of P use efficiency (PUE), P uptake efficiency (PupE) and P utilization efficiency (PutE) were investigated at both normal- P (NP) and low-P (LP) conditions across two environments.

### Data analysis

Phenotypic data was analyzed with SAS 9.0 (SAS Institute Inc., NC, USA) using the GLM procedure. In brief, VARCOMP was used to estimate genotypic variance (σ^2^ G), G × E interaction variance (σ^2^G × E), and error variance (σ^2^E). Broad-sense heritability (*h*^2^) was estimated for each trait according to Hallauer and Miranda (1981):

(1) $$h^{2}\%=\frac{\sigma_{G}^{2}}{\sigma_{G}^{2}+\sigma_{G\times \text{ E}}^{2}/n+\sigma_{E}^{2}/nr}\times 100\%,$$

Where n is the number of batches in each culture system, and r is the mean of the replicates of the different experiments. For each trait, within a specific culture system, the phenotypic value of all replicates from the different batches was performed by the LSMEAN procedure in SAS. This procedure was used for the phenotypic analysis, correlation analysis and QTL mapping. Pearson correlation coefficient and principal coordinate analysis (PCA) were calculated using SPSS Statistics 17.0 (SPSS, Inc., Chicago, IL, USA).

QTL analysis was performed by a composite interval mapping (Zeng, 1994) method using Windows QTL Cartographer version 2.5 (Model 6) (Wang et al., 2005). Forward regression was analyzed using a window size of 10 cM, a walk speed of 2 cM and five control markers. Testing for the presence of a putative QTL in an interval by a likelihood ratio test was performed using a LOD threshold of 2.5. Any loci detected within 11.2 cM (the average interval between each two markers in this population) for each trait, from the different culture systems were considered as the same locus.

## Results

### Evaluation of RSA-related traits in RILs and their parents under three culture systems

**Figure 1:**
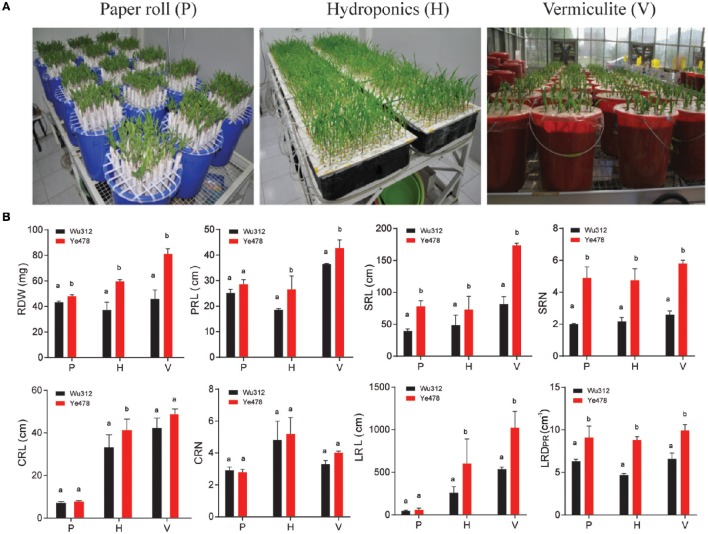
**(A)** Picture representing the three culture systems: Paper roll test (P), Hydroponics (H) and Vermiculite (V). **(B)** The phenotypic difference between the two parental lines Ye478 and Wu312 under the three culture systems. Significant difference between parental lines is indicated by different letters (*P* < 0.05). Abbreviations for root traits are as follows: RDW, root dry weight; PRL, primary root length; SRL, seminal root length; SRN, seminal root number; CRL, crown root length; CRN, crown root number; LRL, lateral root length; LRD_PR_, lateral root density of primary root.

Maize plants were grown in paper rolls (P), hydroponics (H) and a vermiculite (V) culture system (Figure 1A). Eight RSA-related traits were investigated: RDW, PRL, SRL, SRN, CRL, CRN, LRL and LRD_PR_, representing the biomass, length and density of roots (Table 1). Between the two parents, Ye478 had higher RDW, SRL, SRN, LRL, LRD_PR_ than Wu312 in all three culture systems (Figure 1B; Table S1). The increased values in Ye478 compared to Wu312 were to 10.9, 60.6, and 77.1% on RDW; 98, 50.3, and 112.4% on SRL; 145, 118.2, and 123.1% on SRN; 21.3, 133.2, and 89.8% on LRL; and 44.4, 50, and 87.2% on LRD_PR_ in P, H, and V culture system, respectively. However, Wu312 had more CRL and CRN in P and more PRL in V culture system.

Within the RIL population, considerable phenotypic variation existed for eight RSA-related traits (CV values ranged from 9.3 to 64.7%; Table 2) in the three culture systems. Transgressive segregation was also observed for these traits, indicating the presence of multiple genes controlling the investigated traits. Analysis of variances (ANOVA) revealed significant variance in genotype × culture system interaction, indicating strong culture system effects on all investigated root traits (Table 2). Broad-sense heritability (*h*^2^) was calculated for each trait across the three culture systems, which varied from 52.9 to 83.7% (Table 2). LRD_PR_ had the highest *h*^2^ (83.7%), which was followed by SRN (82.6%); while root length had a relatively lower *h*^2^ with 69.1, 68.1, 64.6, and 52.9% for PRL, SRL, CRL, and LRL, respectively.

**Table 2:** **Statistics for the phenotypes of RSA-related traits in RIL population evaluated in paper roll test (P), hydroponics (H), vermiculite (V) culture systems**.

**Trait**	**Culture system**	**Statistical analysis of the RIL population**					**Analysis of Variance^b^**	
		**Median**	**Minimum**	**Maximum**	**CV (%)**	***h^2^* (%)^a^**	**Genotype**	**Genotype × Culture system**
RDW	P	35.6	21	51.2	17.4	75.4	580141.9^**^	323263.3^**^
	H	79.7	51	121.4	22.3			
	V	62.7	33.8	94	19			
PRL	P	28.1	16.8	36.6	13.1	69.1	69414.2^**^	41959.7^**^
	H	26	16.1	35.3	12.5			
	V	40.4	30.6	50.5	9.3			
SRL	P	64.5	27.6	114	24.1	68.4	2162120.3^**^	1169649.6^**^
	H	74.8	31.3	135.7	30.7			
	V	124.9	53.4	205.3	22.1			
SRN	P	3.5	1.3	5.9	23	82.6	5291.7^**^	2007.1^**^
	H	3.3	2.1	6	24			
	V	4.1	1.8	8.3	30.3			
CRL	P	4	0	9.9	64.7	64.6	400226.8^**^	226356.3^**^
	H	31.2	2.6	70.3	42.1			
	V	52	22.9	95.8	27.7			
CRN	P	2.1	0	3.3	40	74.7	2652.6^**^	1468.1^**^
	H	4.8	2.3	7.3	21.6			
	V	3.8	2.3	5.6	15.4			
LRL	P	37.9	2.9	100.8	60.3	52.9	78103129.1^**^	26102207.6^**^
	H	436.2	77.5	815.2	36.8			
	V	711.9	264.7	1250.7	35.2			
LRD_PR_	P	7.6	4.3	11.2	17.2	83.7	12200.6^**^	4364.7^**^
	H	7.2	3.7	14.6	23.6			
	V	6.4	3.5	11.4	23.3			

a h^2^ (%), broad-sense heritability;

b Significant level at P < 0.01 was indicated by ^**^.

**Figure 2:**
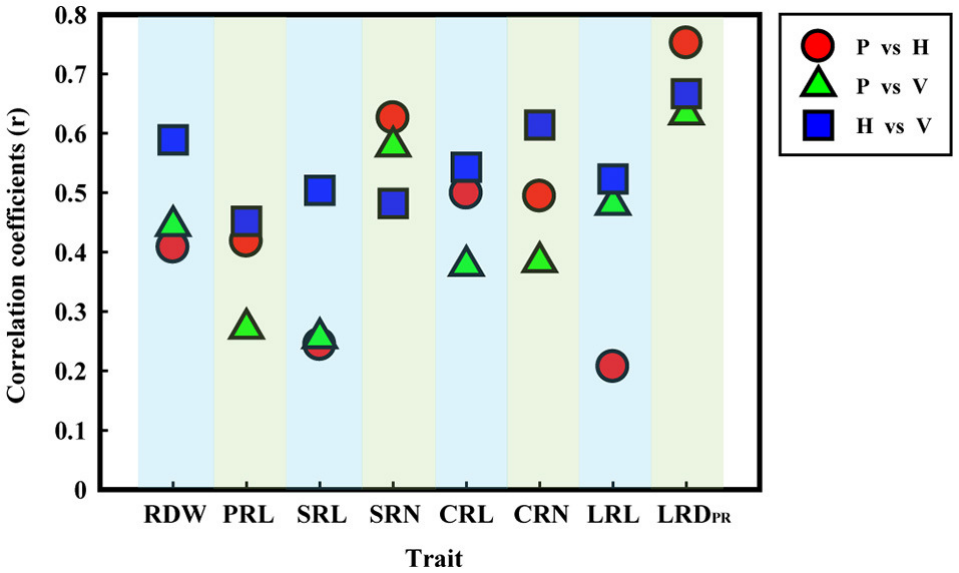
**Diagram of RSA phenotypic correlations among the three culture systems**. Red circles, green triangles and blue rectangles represent the correlation between paper roll test (P) and hydroponics (H), paper roll test (P) and vermiculite (V), and hydroponics (H) and vermiculite (V), respectively.

By the Pearson correlation analysis, a close relationship was observed for each RSA trait among the different culture systems (Figure 2). In general, the average correlation coefficients showed the highest level (*r* = 0.55) between H and V; medium level (*r* = 0.46) between P and H; and the lowest level (*r* = 0.43) between P and V culture system (Figure 2; Table S2). The following RSA traits, RDW, PRL, SRL, CRL, CRN, and LRL (except SRN and LRD_PR_) showed a higher correlation between H and V system as compared to the P system. The coefficient values were *r* = 0.59, 0.45, 0.50, 0.54, 0.61, and 0.52, respectively. However, the coefficients of SRN and LRD_PR_ were higher between the P and H system with values of *r* = 0.63 and *r* = 0.75, respectively. Moreover, no significant correlation was found for SRL and LRL between the P and H culture systems, and for SRL in the P and V systems.

### Phenotypic relation between RSA-, nutrient efficiency-related traits

Since RSA contributes directly to plants nutrition acquisition, we analyzed the correlations between RSA and nutrient efficiency traits within this population. The nutrient related traits were generated from field experiments, which included NUE, NupE and NutE in different nitrogen treatments (Li et al., 2015); and PUE, PupE, PutE in different phosphorous treatments (Gu et al., 2016). No significant correlation was found between N or P efficiency related traits and RSA traits grown in the P culture system (Tables S3, S4). However, significant correlations were observed between nutrient uptake efficiency and RSA traits with plants grown in the H or V culture systems. SRL from the H and V system showed significant positive correlation to N efficiency traits irrespective to nitrogen treatments: including LN-NUE (*r* = 0.19–0.20), HN-NupE (*r* = 0.17–0.22) and LN-NupE (*r* = 0.21–0.24). SRN for the H and V culture showed similar correlation LN-NupE (*r* = 0.24–0.31). CRL from the H system, but not from the P and V systems, had significant positive correlations (*r* = 0.20 and 0.19) to HN-NUE and HN-NupE, respectively. Significant negative correlations were observed only between RSA and NutE traits. LRL from both the H and V systems showed significant correlations to HN-NutE with *r* = −0.20 and −0.24, respectively. Negative correlations between V cultured RDW and NutE in both HN and LN conditions were also found with *r* = −0.19 and −0.20, respectively.

Correlations between the H and V cultured RSA and P efficiency related traits were also observed from this population. SRL from H systems had significant positive correlation to LP-PUE (*r* = 0.25) and NP-PupE (*r* = 0.22) (Table S4). SRL from the V system also positively correlated to LP-PUE (*r* = 0.27) and LP-PutE (*r* = 0.18). Besides SRL, RDW from the H and V systems had positive correlations to LP-PupE. Related to LR traits, only LRL from V system showed the highest positive correlation to LP-PupE (*r* = 0.34). However, LRD_PR_ had negative correlations to LP-PupE and LP-PutE (*r* = −0.21 and −0.20, respectively).

**Figure 3:**
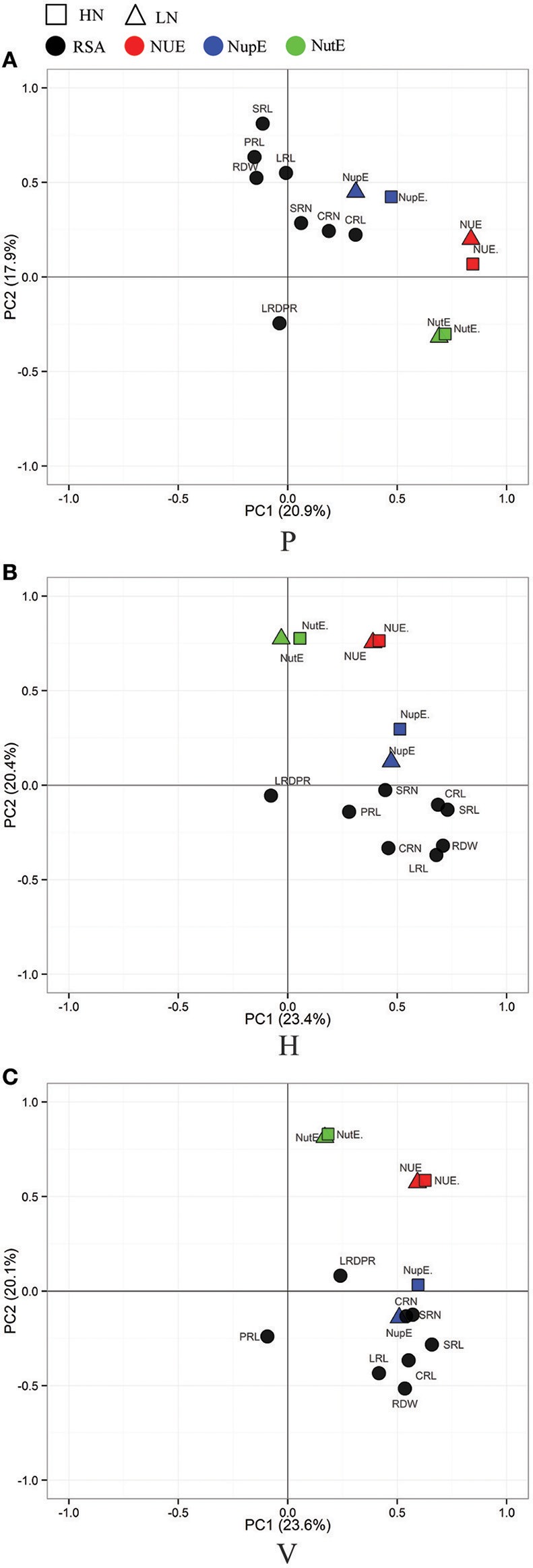
**Principal Component Analysis (PCA) of the RIL population for RSA- (in black) and N efficiency related trait**. RSA traits were evaluated in three culture systems of paper roll test **(A)**, hydroponics **(B)**, and vermiculite **(C)** culture and N efficiency related traits (NUE, red; NupE, blue; NutE, green) were evaluated in the field under the conditions of high-nitrogen (HN, in rectangle) and low-nitrogen (LN, in triangle). NUE, nitrogen use efficiency; NupE, nitrogen uptake efficiency; NutE, nitrogen utilization efficiency.

**Figure 4:**
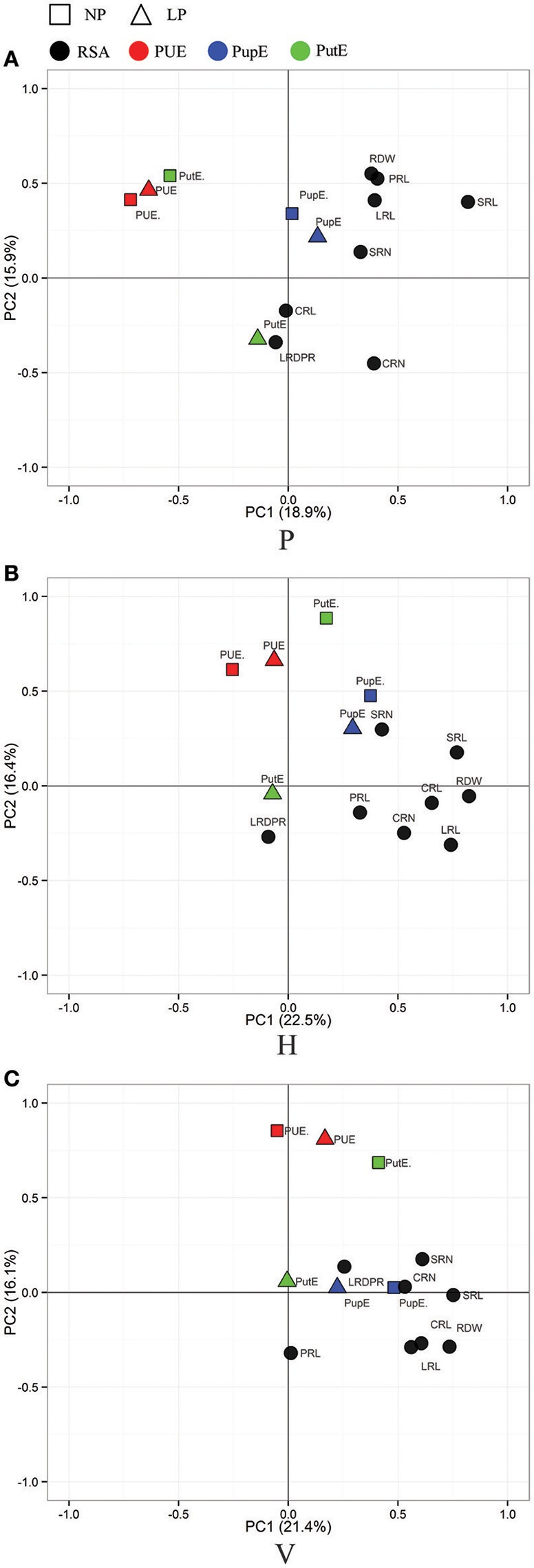
**Principal Component Analysis (PCA) of the RIL population for RSA-related traits (in black) and P efficiency related traits**. RSA traits were evaluated in three culture systems of paper roll test **(A)**, hydroponics **(B)**, and vermiculite **(C)** culture. P efficiency related traits (PUE, red; PupE, blue; PutE, green) were evaluated in the field under the conditions of normal-phosphorus (NP, in rectangle) and low-phosphorus (LP, in triangle). PUE, phosphorus use efficiency; PupE, phosphorus uptake efficiency; PutE, phosphorus utilization efficiency.

A principle component analysis (PCA) was performed to visualize the correlation between the three culture generated RSA traits and nutrient efficiency traits (Figures 3, 4). As shown in Figure 3, root traits (expect LRD_PR_) from the three culture systems were all closely related with LN-NupE and HN-NupE, thus indicating a closer relationship of the maize root to nitrogen uptake rather than to nitrogen utilization. Among the three culture systems, the first two principal coordinates explained 44.3 and 43.7% of the total variance in the H and V system, respectively; while only 38.8% explained in the P system. Analogously, root traits (except LRD_PR_ in H, PRL in V, and CRL and LRD_PR_ in P) that were closely related to LP- and HP-PupE, explained 38.9, 37.5, and 33.9% of the total variance under the H, V, and P culture systems, respectively. Irrespective of N and P levels, the correlation between root traits and nutrient uptake efficiency traits was higher in H and V than that in the P system. These results suggest that the H and V culture systems are more suitable for the investigation of RSA and nutrient uptake efficiency related traits than the P system.

### Detection of QTLs for RSA-related traits

Forty-six putative QTLs were detected in the RIL population for 8 root traits with 14, 18, and 14 from the P, H, and V culture system, respectively (Table 3). Within the identified QTLs, a similar proportion of QTLs carried the favorable allele that originated from either the parental line Ye478 or Wu312. Phenotypic variation explained by each QTL for root-related traits ranged from 5.9 to 40.3% (Table 3). No common QTL could be detected from all three culture systems, and three common QTLs were repeatedly identified from two of the three systems (Table 3). Two common QTLs for SRL and CRL were repeatedly detected from the H and V systems that localized at chromosomal bin3.04 and bin1.06/1.07, respectively. The QTLs for SRL had favorable alleles from Ye478 in both culture systems, and explained a phenotypic variation of 9.7 to 10.1%. Favorable alleles from Wu312 were observed for the CRL common QTLs. The last common QTL was a LRD_PR_ QTL that was repeatedly detected from the P and V culture systems, and located at bin5.04/5.05 that explained 7.2–25.8% of the phenotypic variation.

**Table 3:** **Summary of the detected QTLs for RSA-related traits from RIL population grown in the paper roll test (P), hydroponics (H) and vermiculite (V) culture systems**.

**Trait**	**Chromosome**	**Bin^a^**	**Position**	**Interval**	**Flanking marker**		**R^2,^^b^**	**Additive^c^**	**LOD**		
									**P**	**H**	**V**
RDW	3	3.04	117	115–119	umc1504	umc1773	5.9	3	0.5	0.5	**2.6^d^**
	3	3.04	73	70–78	umc1012	phi029	17.3	3.8	0.3	**4.9**	0.1
	5	5.04–5.05	136	125–154	umc1221	umc2164	20.28	−4.10	0.6	**2.8**	0.5
	7	7.03	108	103–114	mmc0411	umc1888	9.76	3.22	**2.9**	0	1.9
PRL	2	2.02–2.03	34	25–42	umc1542	bnlg2248	8.9	−1.0	0.3	**3.7**	0.1
	3	3.04	120	117–125	umc1223	phi035	9.6	1.2	**2.8**	0.3	1
	4	4.05	84	76–89	umc1662	umc1953	12.3	−1.2	0.1	**4.3**	0.4
	5	5.03	34	22–40	bnlg1879	umc1447	9.8	−1.0	0.8	**3.2**	1.3
	5	5.07	225	216–228	phi048	umc1072	9.7	1.2	**2.8**	0.1	1
SRL	2	2.05	84	78−89	umc1635	umc1003	15.5	−6.2	**3.2**	0.4	0.6
	3	3.04	69,74	69−79	umc1012	phi029	12.7,11.8	10.1,9.7	2.2	**4.6**	**4.2**
	8	8.07–8.08	135	126−147	umc1268	umc1933	12.5	8.2	1.0	**2.8**	0.3
	9	9.01	6	0−16	umc1867	bnlg1583	9.5	7.3	0.2	**2.8**	0.3
SRN	2	2.01–2.02	10	4−19	phi96100	umc1518	7.1	0.3	0.6	0.4	**3.2**
	2	2.06–2.07	107	106−111	nc003	umc1637	14.5	0.4	0	**3.6**	0.6
	2	2.07	122	118−131	umc1637	bnlg2077	9.8	0.3	0	**3.2**	0.3
	5	5.07–5.09	239	233−241	umc1072	bnlg386	17.8	−0.3	**2.9**	0.1	0
	6	6.05	92	86−95	umc1020	bnlg1732	19.3	0.4	**3.3**	0	0.1
	6	6.05–6.07	114	113−123	bnlg1732	umc2165	19.3	−0.4	**4.6**	0.1	0.4
CRL	1	1.02–1.03	73	66−75	umc1568	umc1403	19.5	−7.0	0.5	**3.7**	0.4
	1	1.06–1.07	160, 169	151−174	umc1754	bnlg1556	12.5,31.9	−4.9, −8.4	0.1	**2.7**	**4.1**
	3	3.01	9	2−18	umc1793	umc1394	8.3	4.2	0	0.8	**2.9**
	4	4.05–4.06	124	113−142	umc1346	bnlg2291	20.9	−1.2	**2.7**	0.3	0.2
	5	5.04	59	52−68	phi113	umc1990	7.4	−4.0	0.6	0.1	**2.8**
	6	6.07	167	149−175	phi299852	bnlg1740	16.7	6.1	0.1	0.4	**2.9**
	8	8.06–8.07	107	98−113	umc1997	bnlg1823	16.2	−5.5	0.1	**3.8**	0
	8	8.09	195	190−201	phi015	bnlg1131	7.2	−3.9	0.4	0.1	**3.1**
CRN	1	1.04–1.05	128	122−131	umc2112	bnlg1884	15.0	0.5	0.8	**4.4**	0.1
	1	1.06	152	149−160	umc1754	umc1335	21.3	−0.5	1.3	**4.7**	2.1
	4	4.04–4.05	74	71−79	umc1963	phi079	14.5	0.4	**2.8**	0	0.1
	6	6.04–6.05	61	50−66	umc1857	bnlg1174	7.4	0.3	0.9	**2.5**	0
	7	7.03–7.04	140	137−152	umc1301	umc1710	19.4	−0.4	**3.2**	0.2	0
LRL	4	4.05	78	68−87	umc1662	phi079	10.2	−53.0	1.2	**3**	0.8
	5	5.01–5.03	32	18−36	bnlg1879	umc1447	10.4	−7.5	**3.3**	0.3	0.9
	5	5.05	152	133−159	umc2164	bnlg278	29.8	−137.9	0.2	2.4	**3.4**
	3	3.08	211	205−217	phi046	umc1320	43.8	166.6	1.1	0.1	**3.5**
	3	3.08–3.09	234	228−241	umc1320	phi047	45.0	169.5	0.6	2	**3.5**
	10	10.03	58	53−66	umc1345	umc1336	49.9	183.0	0.4	0.7	**4.1**
LRD_PR_	1	1.05–1.06	140	128−161	bnlg1884	umc1754	15.6	0.7	3	**2.7**	2.3
	1	1.06	155	149−173	umc1754	umc1335	15.1	0.5	**3**	2.5	0.3
	2	2.04–2.05	74	67−78	umc2248	umc1003	7.2	0.4	0.3	0.1	**3.4**
	4	4.07	149	146−154	umc1620	umc1194	25.8	−0.7	**5.6**	1.2	0.5
	5	5.04–5.05	144, 150	133−161	umc1221	bnlg278	10.3,19.8	−0.5, −0.6	**6**	2.3	**2.7**

a Chromosome bins of the marker and position taken from IBM 2008;

b R^2^, the percentage of the phenotypic variance explained by a putative QTL;

c Add, additive effects; Positive and negative values represented Ye478 and Wu312, respectively, that carried the allele for an increase of trait value;

d *Bold number represented the LOD value for a detected QTL in the corresponding culture system*.

Besides these three common QTLs, another 40 QTLs were identified as single QTLs that were detected from only one culture system. Two QTLs were identified for SRL from both the P and H culture with an explained variance ranging from 9.5 to 15.5%. Six and four environmental QTLs were found for CRL and LRD_PR_, respectively. Two QTLs for PRL were identified from the P culture with a total explained variance of 19.3%. Another three PRL QTLs were identified from the H culture system with a total explained variance of 30.9%. These five PRL QTLs were located on chromosomes 2, 3, 4, and 5, with about half of the favorable alleles from Ye478 or Wu312. Three, two and one QTLs were identified for SRN from the P, H, and V system, respectively. The three P environmental QTLs explained a total phenotypic variance of 56.4% and were located on chromosome bin5.07/5.09, 6.05, and 6.05–6.07. For CRN, three QTLs were detected from the H environment with a total explained variance of 43.7%; another two CRN QTLs were from the P environment, and no QTL was detected from the V environment. Four QTLs were identified for RDW with two from H, and each one from the P and V environments. Six QTLs for LRL were identified with one each from P, H and four from the V environment.

### Co-location of QTLs for RSA-related traits

**Figure 5:**
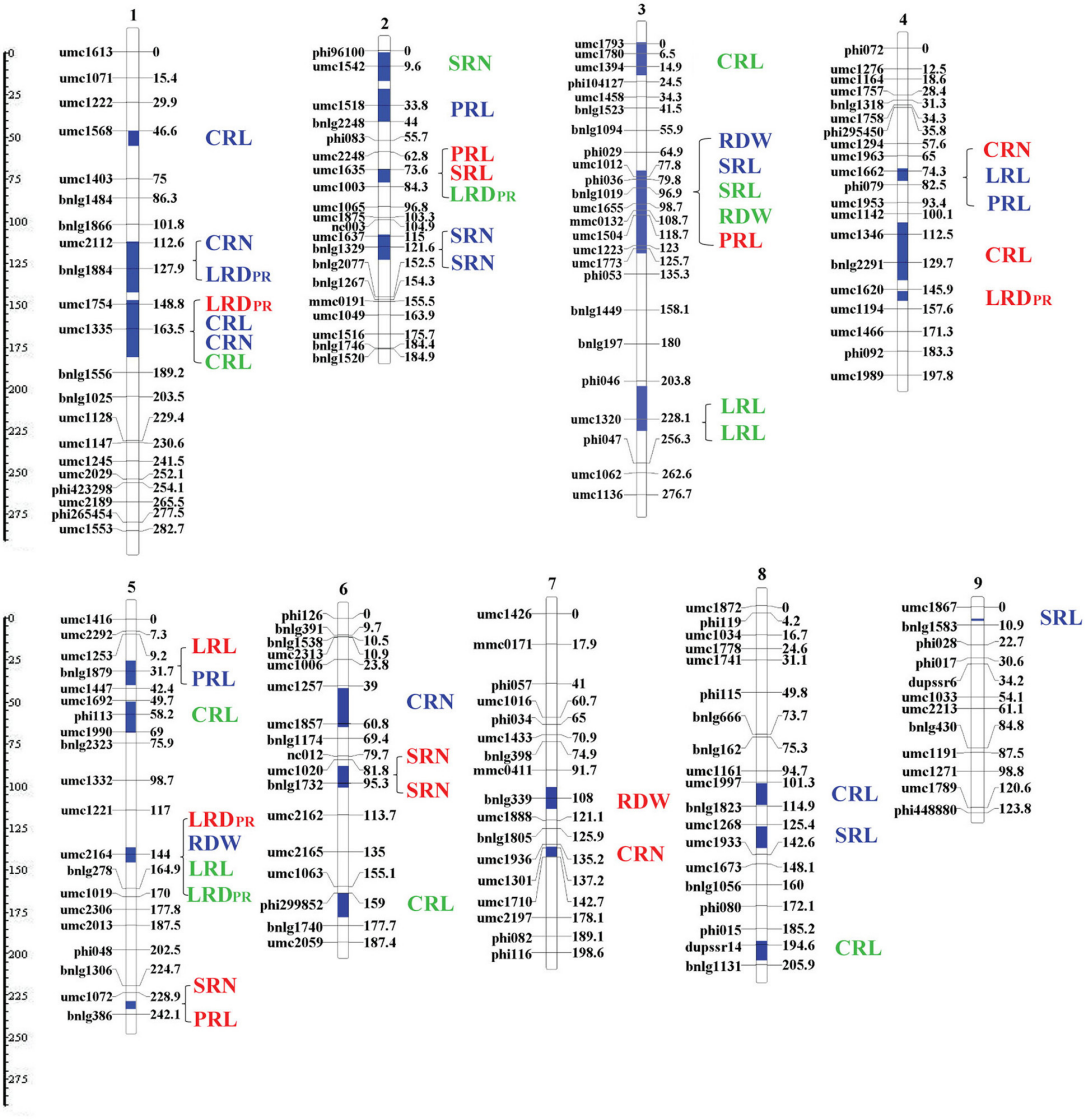
**Location of the QTL clusters detected for all investigated traits as revealed by meta-QTL analysis**. QTL clustering determined by MetaQTL software as described by Veyrieras et al. (2007). The vertical blue lines in chromosomes represent the maker interval where QTLs were located. Each boxplot represents the identified QTL cluster, within which the QTL name was presented on the right. QTLs identified from paper roll, hydroponics and vermiculite were marked by red, blue and green letters, respectively.

Co-localization of QTLs for different RSA traits were majorly located in six chromosome regions of bin 1.04/1.05, 1.06, 2.04/2.05, 3.04, 4.05, and 5.04/5.05 (Figure 5). The most noteworthy overlaps occurred on chromosomal bin3.04 where five QTLs are located. Three QTLs were co-localized between umc1012 and phi029: two associated with SRL and RDW from the H culture system and one with SRL from V culture. In a nearby chromosome region, two QTLs were located with one (umc1504–umc1773) associated with RDW from the V system and the other (umc1223-phi035) associated with PRL from P culture. On chromosomal bin1.06, four QTLs were co-localized between umc1754 and umc1335/bnlg1556: two QTLs associated with CRN and CRL from the H culture; one each associated with LRD_PR_ from P culture and CRL from V culture. In addition, a QTL of LRD_PR_ from the H culture was located in a nearby region (bnlg1884-umc1754). Two QTLs, one associated with CRN from the H culture and the other for LRD_PR_ from the H system were co-localized on chromosome bin1.04/1.05 (umc2112-bnlg1884). On chromosome bin2.04/2.05, two QTLs were co-localized between umc2248 and umc1003: one QTL for SRL from H culture, and one QTL for LRD_PR_ from V culture. One overlap of QTLs for PRL, LRL in H culture and CRN in P culture was co-localized on chromosome bin4.05. An overlap of QTLs for root traits also occurred on bin5.04/5.05, which associated with LRL and LRD_PR_ QTLs from V culture, and RDW QTL from H and LRD_PR_ QTL from P culture.

## Discussion

### RSA performance in paper roll, hydroponics and vermiculite systems

The maize root system is crucial for plant establishment as well as water and nutrient uptake. Substantial genetic and phenotypic variations were found for root architecture, providing an opportunity for genetic research (Burton et al., 2013). Numerous amount of work has been conducted to dissect the genetic mechanisms that control RSA traits under various growth conditions: hydroponics and paper roll (Lebreton et al., 1995; Hoecker et al., 2006; Zhu et al., 2006). However, results from this work were inconsistent. Comparisons of root performance between the differing growth conditions could provide a better understanding of the genetic basis of RSA and ultimately identify stable QTLs for future map-based cloning and marker associated selection. In order to gain a better understanding of the effects of culture systems on RSA traits and to discover an ideal culture system for the genetic research of these traits, we performed extensive phenotyping of maize seedlings grown in H, V and P culture systems.

Notable differences were present between the three culture systems for RDW, PRL, SRL, CRL, and LRL (Table 2). The H system was characterized by a higher RDW compared to the other two systems. However, other root traits (except CRN and LRD_PR_) in the H system were lower than that in the V system. Primary and seminal roots are embryogenic roots that make up a major portion of the root biomass in the first few weeks of maize seedling growth (Hochholdinger and Tuberosa, 2009). The higher RDW, but lower PRL, SRN, and SRL indicated a bigger root diameter in the H system that may have resulted in bigger RDW without having a big root number and/or length (Table 2). Moreover, RDW showed higher correlations (*r* = 0.41–0.59) between the two culture systems compared to PRL (0.27-0.45) and SRL (0.24–0.50) (Table S2). Paired with the fact that RDW at the seeding stage showed the closest relationship with final grain yield (Cai et al., 2012), suggests that RDW might be a more reliable indicator for genetic evaluation of maize RSA at early growth stages.

The range of values of SRN (2–8) was relatively consistent across all three systems in the present study (Table 2). This was similar to a previous observation of 2.2–8.4/plant in greenhouse (Hund et al., 2004), 0–8/plant in cigar rolls (Hoecker et al., 2006; Zhu et al., 2006), and 0–6/plant in field (Bayuelo-Jimenez et al., 2011). In addition, strong trait correlations (*r* = 0.48–0.63) in the different culture systems and a high heritability (82.6%) were observed for SRN (Table 2 and Figure 2), suggesting that SRN was a stable RSA trait under the different growth conditions with minimal environmental effect.

The lateral root density of maize has been extensively investigated, as a developmentally meaningful parameter. An increased lateral root density may significantly contribute to early seedling vigor (Hoecker et al., 2006; Paschold et al., 2010). In this study, the lateral root density of the primary root (LRD_PR_) is equal to the number of emerged roots divided by the length of the branching zone of the primary root. The results show that LRD_PR_ has a considerably higher heritability (83.7%) and lower CV% under the three culture systems (Table 2). Moreover, in the different culture systems, LRD_PR_ was significantly correlated with the highest correlation coefficients ranging from 0.63 to 0.75 (Figure 2), indicating that LRD_PR_ may be another stable root system trait.

Lateral roots typically account for a major portion of the root system of vascular plants (Husakova et al., 2013) and are responsible for the major acquisition of nutrients and water (McCully and Canny, 1988; Postma et al., 2014). LRL was an integrative result of lateral root initiation, emergence and elongation (Dubrovsky and Forde, 2012), and could be easily affected by both abiotic and biotic factors (Lynch, 2013). So it was not surprising to find LRL to be highly variable (60.3, 35.2, and 36.8% CV in paper roll, hydroponics and vermiculite culture, respectively; Table 2). Therefore, the results indicate that the LRL was more easily affected by the environments.

### Correlation of RSA and nutrient related traits

In general, nutrient use efficiency refers to the ability of plants to produce biomass or yield under certainly available nutrient conditions. It is further divided into two main components: nutrient uptake efficiency, the ability of plants to acquire nutrients from the soil; and nutrient utilization efficiency, the ability of plants to use available nutrients to increase grain yield (Moll et al., 1982; Lynch, 1998; Wissuwa, 2003; Wang et al., 2010). Enhancing the nutrient use efficiency in plants can be achieved by improving either the uptake or utilization of nutrients or by improving both. In this study, the phenotypic correlation and PCA showed that RSA among the different growth conditions had significant associations with nutrient efficiency related traits. Importantly, RSA had a positive correlation with PupE and NupE between the H and V systems, but no correlation was found from the P culture system (Figures 3, 4). The differing correlations can be explained by the particular characteristics of the three culture systems. Due to the physical limitations in the P system, three-dimensional root growth was expected to be restricted. Additionally, the diffusional resistance for oxygen and lower O2 activities in the root zone increased in the roll system. No significant correlation between RSA-related traits in the P system and PupE, NupE was found as a result of these factors. The plants grown in the H system have a uniform supply of nutrients and a lower mechanical resistance. This can enhance the correlation between RSA-related traits and NupE in the H system under N deficiency conditions (Table S3). However, the V system better simulates actual field conditions by providing mechanical resistance. The poor mobility of nutrients in the V system, caused the plants to increase root growth in order to exploit more nutrients. For example, the root trait LRL had a significant increase in V system compared to H and P systems with the highest correlation value with PupE found in the LP condition.

Significant correlations between nutrient uptake efficiency (NupE and PupE) and RSA traits were observed when plants were grown in the H and V culture systems. Although the correlation coefficient values were weak, such as HN-NupE (*r* = 0.17–0.22) and LN-NupE (*r* = 0.21–0.31) (Table S3). A similar study has shown that the length and number of seminal root in hydroponics have weak positive correlations with NupE (*r* = 0.15–0.31) in the same RIL population under HN and LN conditions (Li et al., 2015). RDW, SRL, and LRL under LP conditions, seem to be more relevant to PupE (*r* = 0.22–0.34) (Table S4). In the same bi-parental population, a significant positive correlation between RDW, SRL in hydroponics and PupE (*r* = 0.25–0.30) was observed (Gu et al., 2016). Using a diverse set of 74 inbred Maize lines, Abdel-Ghani et al. (2013) observed that SRL of maize seedlings have weak significant correlations to grain yield under HN (*r* = 0.36) and LN (*r* = 0.24) levels. Additionally, under drought conditions, Tuberosa et al. (2002b) also found a weak relationship between RSA and grain yield (*r* = 0.2–0.3). These findings indicate that the genetic correlation between RSA and nutrient uptake efficiency, and grain yield really exists. A genetic relationship between RSA and nutrient uptake efficiency is essential for breeders to consider RSA as a selection criterion to improve nutrient efficiency in any maize breeding program. However, it should be noted that breeding could be affected by the low RSA correlation. Given the time and labor constraints in extracting intact root systems from the field, it is not easy to capture maize RSA traits from large numbers of genetic material under field conditions. RSA traits at the seedling stage in indoor culture systems are simple to analyze and can be used to guide the practice of developing high nutrient uptake efficiency genotypes. In this study, the H and V systems appear to be better for selection of RSA traits. Additionally, the V system was the best option for pursuing LRL and LPDPR research, and the H system for SRL and SRN research under nutrient stress. (Figures 3, 4, Tables S3, S4). From this point of view, there is still a certain value of the application. Given that the direct selection of RSA traits under field conditions is the best way to breed for N and P efficiency varieties, more advanced higher throughput methods with a more accurate phenotypic analysis of RSA field traits should be created for future breeding practices.

### QTL stability for root trait across different culture systems

Stable QTLs identified under differing environments were beneficial for the improvement of targeted traits via marker-assisted selection (MAS) and for cloning of underlying candidate genes (Cai et al., 2012). In this study, six out of the total 46 QTL (13%) were repeatedly detected from two culture systems (Table 3). The six common QTLs detected in this work belonged to SRL, LRD_PR_, and CRL traits. The high broad-sense heritability trait SRN, was not repeatedly detected under the different growth conditions. Through the extensive phenotyping of a mapping population, we have clearly shown that seedling root traits in maize interact strongly with the growth environment in all three culture systems. Although growth conditions were similar for both the H and P systems, the P systems physical limitations and low oxygen environment restricted root growth. The V system in the greenhouse had different light, temperature and humidity conditions as compared to the H and V systems in the growth chamber. Thus, strong genetic-environmental interactions were expected which resulted in few common repeatably identifiable QTLs. The rarity of detecting repeated QTLs for RSA traits from the different environment was also published in previous works. Hund et al. (2004) and Tuberosa et al. (2002a) used two different culture systems of sand/vermiculite and hydroponics, but only detected seven common QTLs.

The RSA QTLs detected in this work were distributed throughout the maize chromosome mainly in five putative regions (bins1.06, 2.04, 3.04, 4.05, and 5.04) (Table 3). A region on chromosome 3 (bin3.04) was the most noteworthy region in that, it included five QTLs for RSA traits for RDW, PRL, and SRL, respectively (Figure 5). The two QTLs for SRL and RDW were detected in the H and V systems, respectively. Several previous studies also indicated that bin 3.04 is an important chromosome region for root traits. In this region QTLs were identified for RSA and NupE and PupE (Li et al., 2015; Gu et al., 2016), as well as yield-related traits under different environmental conditions (Agrama et al., 1999; Qiu et al., 2007; Liu et al., 2008; Messmer et al., 2009). In previous studies, the two identified chromosomal regions bin 1.06 and bin 2.04 were also reported to affect RSA. QTLs in bin 1.06 have been shown to influence primary root length, primary root diameter, primary root weight and root volume in different mapping populations (Kaeppler et al., 2000; Tuberosa et al., 2002a). This region was also associated with maize root adaptation to abiotic stress, such as low nitrogen stress (Liu et al., 2008, 2011) and low phosphorus stress (Zhu et al., 2006; Chen et al., 2008). QTLs in bin 2.04 have been reported as simultaneously affecting both plant roots and above-ground traits (Tuberosa et al., 1998). Thus, these three QTL regions control maize RSA traits, which could be promising candidates for cloning the underlying genes for improving maize root system, as well as water, nitrogen and phosphorus use efficiency.

## Conclusion

A number of approaches relying on plants grown in hydroponics, vermiculite and paper rolls have been used for evaluating root traits (Tuberosa et al., 2002b; Hund et al., 2004; Zhu et al., 2006). Although each approach could provide an accurate RSA evaluation under controlled environmental conditions, a comparison of RSA performance under the different culture systems was unclear. From this work, a greater phenotypic variation of RSA was observed when plants were grown in hydroponics and vermiculite; with a significant correlation for each RSA trait observed between these two growth conditions. The RSA from the paper roll seemed to be different, which showed lower or no significant correlation of RSA to the H and V systems. Furthermore, RSA-related traits generated from the hydroponics and vermiculite system, but not from the paper roll, showed significant positive correlation to nutrient (nitrogen and phosphorus) uptake efficiency traits. Although the correlations between RSA and nutrient uptake efficiency traits were weak, some true genetic correlation exists. It is essential to consider RSA when using H and V systems in the seedling stage as an important index to develop highly nutrient uptake efficient genotypes in maize breeding programs. When not having to care about the costs, direct selection for RSA under field conditions would be the best way to breed for N and P efficient varieties.

## Author contributions

GM and FC conceived and designed the experiments. KG and SS performed the experiments. ZL analyzed the data. ZL, SS, and RG drafted the manuscript, which was reviewed by LY, ZW, and FC. The revised version was edited by EC.

### Conflict of interest statement

The authors declare that the research was conducted in the absence of any commercial or financial relationships that could be construed as a potential conflict of interest.
