# Supplementary material for: Comparative Analysis of Root Traits and the Associated QTLs for Maize Seedlings Grown in Paper Roll, Hydroponics and Vermiculite Culture System
Source: Front Plant Sci. 2017 Mar 30;8:436. doi: 10.3389/fpls.2017.00436 (PMC5371678; doi:10.3389/fpls.2017.00436)
Supplement: Supplementary file 1 [file Table1.DOCX]

**SUPPLEMENTARY DATA**

**Comparative analysis of root traits and the associated QTLs for maize seedlings grown in paper roll, hydroponic and vermiculite culture system**

Zhigang Liu^1^, Kun Gao^1^, Shengchen Shan^1^, Riling Gu^1^, Zhangkui Wang^1^, Eric J. Craft^2^, Guohua Mi^1^, Lixing Yuan^1^ and Fanjun Chen^1^*

*^1^ Key Lab of Plant-Soil Interaction, MOE, Center for Resources, Environment and Food Security, College Resources and Environmental Sciences, China Agricultural University, Beijing, China*

*^2^ USDA-ARS, Robert Holley Center for Agriculture and Health, 538 Tower Road, Ithaca, NY 14580, USA.*

* Correspondence:

Fanjun Chen, Key Lab of Plant-Soil Interaction, MOE, Center for Resources, Environment and Food Security, College Resources and Environmental Sciences, China Agricultural University, Beijing, 100193, China

E-mail: [caucfj@cau.edu.cn](mailto:caucfj@cau.edu.cn)

Tel: 86-010-62734454; Fax: 86-010-62731016

**Table S1.** Statistics for RSA related traits of the parent lines across three culture systems. Statistics for root dry weight (RDW), primary root length (PRL), seminal root length (SRL), seminal root number (SRN), crown root length (CRL), crown root number (CRN), lateral root length (LRL), lateral root density of primary root (LRD_PR_) of the parent lines grown in paper roll test (P), hydroponics (H) and vermiculite (V) culture systems, respectively.

**Table S2.** Pearson’s correlation coefficients of RSA-related traits evaluated in the paper roll test (P), hydroponics (H) and vermiculite (V) culture systems, respectively.

**Table S3.** Pearson’s correlation coefficients of RSA-related traits in three culture systems and N efficiency related trait in the field evaluated under high-nitrogen (HN) and low-nitrogen (LN) levels. Nitrogen use efficiency (NUE), nitrogen uptake efficiency (NupE), nitrogen utilization efficiency (NutE).

**Table S4.** Pearson’s correlation coefficients of RSA-related traits in three culture systems and P efficiency related trait in the field evaluated under normal- phosphorus (NP) and low- phosphorus (LP) levels. Phosphorus use efficiency (PUE), phosphorus uptake efficiency (PupE), phosphorus utilization efficiency (PutE).

**Table S1.** Statistics for RSA related traits of the parent lines across three culture systems. Statistics for root dry weight (RDW), primary root length (PRL), seminal root length (SRL), seminal root number (SRN), crown root length (CRL), crown root number (CRN), lateral root length (LRL), lateral root density of primary root (LRD_PR_) of the parent lines grown in paper roll test (P), hydroponics (H) and vermiculite (V) culture systems, respectively.

| Culture systems | Trait | Ye478 | Wu312 | % of Increase^1^ |
| --- | --- | --- | --- | --- |
| Paper roll test (P) | RDW | 47.9^a2^ | 43.2^b^ | 10.9 |
|  | PRL | 28.5^a^ | 25.1^a^ | 13.5 |
|  | SRL | 78.2^a^ | 39.5^b^ | 98 |
|  | SRN | 4.9^a^ | 2^b^ | 145 |
|  | CRL | 7.7^a^ | 7.2^a^ | 6.9 |
|  | CRN | 2.8^a^ | 2.9^a^ | -3.4 |
|  | LRL | 55.3^a^ | 45.6^a^ | 21.3 |
|  | LRD_PR_ | 9.1^a^ | 6.3^b^ | 44.4 |
| Hydroponics (H) | RDW | 59.6^a^ | 37.1^b^ | 60.6 |
|  | PRL | 26.6^a^ | 18.5^b^ | 43.8 |
|  | SRL | 73.2^a^ | 48.7^b^ | 50.3 |
|  | SRN | 4.8^a^ | 2.2^b^ | 118.2 |
|  | CRL | 41.2^a^ | 33.2^b^ | 24.1 |
|  | CRN | 5.2^a^ | 4.8^a^ | 8.3 |
|  | LRL | 602.7^a^ | 258.4^b^ | 133.2 |
|  | LRD_PR_ | 9.9^a^ | 6.6^b^ | 50 |
| Vermiculite (V) | RDW | 81.1^a^ | 45.8^b^ | 77.1 |
|  | PRL | 42.7^a^ | 36.5^b^ | 17 |
|  | SRL | 173.5^a^ | 81.7^b^ | 112.4 |
|  | SRN | 5.8^a^ | 2.6^b^ | 123.1 |
|  | CRL | 48.8^a^ | 42.3^a^ | 15.4 |
|  | CRN | 4^a^ | 3.3^a^ | 21.2 |
|  | LRL | 1022^a^ | 538.4^b^ | 89.8 |
|  | LRD_PR_ | 8.8^a^ | 4.7^b^ | 87.2 |

^1^ % of Increase on Mean = (Ye478-Wu312)/Wu312×100%;

^2^ Significant difference between two genotypes in the same culture system was indicated by different letters (P<0.05).

**Table S2**. Pearson’s correlation coefficients of RSA-related traits evaluated in the paper roll test (P), hydroponics (H) and vermiculite (V) culture systems, respectively.

| Trait | Correlation coefficients between different culture systems | | |
| --- | --- | --- | --- |
|  | P vs H | P vs V | H vs V |
| RDW | 0.41** | 0.44** | 0.59** |
| PRL | 0.42** | 0.27** | 0.45** |
| SRL | 0.24 | 0.26 | 0.50** |
| SRN | 0.63** | 0.58** | 0.48** |
| CRL | 0.50** | 0.38** | 0.54** |
| CRN | 0.49** | 0.38** | 0.61** |
| LRL | 0.21 | 0.48** | 0.52** |
| LRD_PR_ | 0.75** | 0.63** | 0.67** |

* and ** indicated significant correlation at *P* <0.05 and 0.01, respectively.

**Table S3**. Pearson’s correlation coefficients of RSA-related traits in three culture systems and N efficiency related trait in the field evaluated under high-nitrogen (HN) and low-nitrogen (LN) levels. Nitrogen use efficiency (NUE), nitrogen uptake efficiency (NupE), nitrogen utilization efficiency (NutE).

| Culture systems | Trait | RDW | PRL | SRL | SRN | CRL | CRN | LRL | LRD_PR_ |
| --- | --- | --- | --- | --- | --- | --- | --- | --- | --- |
| P | HN-NUE | 0.08 | -0.06 | 0.11 | 0.09 | 0.23 | 0.09 | 0.09 | -0.04 |
|  | LN-NUE | -0.02 | 0.02 | -0.07 | 0.02 | 0.09 | 0.07 | -0.06 | -0.09 |
|  | HN-NupE | -0.02 | 0.10 | 0.07 | -0.07 | 0.20 | 0.09 | 0.16 | -0.04 |
|  | LN-NupE | 0.01 | -0.03 | 0.13 | 0.11 | 0.19 | 0.12 | -0.04 | -0.19 |
|  | HN-NutE | -0.12 | -0.19 | -0.13 | 0.12 | 0.06 | -0.09 | -0.01 | 0.10 |
|  | LN-NutE | -0.11 | -0.07 | -0.22 | -0.06 | 0.01 | 0 | 0.04 | -0.07 |
| H | HN-NUE | 0.03 | 0.02 | 0.11 | 0.09 | 0.20^*^ | -0.06 | -0.01 | -0.09 |
|  | LN-NUE | 0.04 | -0.05 | 0.19^*^ | 0.13 | 0.15 | -0.06 | 0.04 | -0.01 |
|  | HN-NupE | 0.17 | 0.12 | 0.17^*^ | 0.15 | 0.19^*^ | -0.04 | 0.12 | -0.13 |
|  | LN-NupE | 0.11 | -0.07 | 0.24^**^ | 0.31^**^ | 0.17 | -0.02 | 0.09 | -0.07 |
|  | HN-NutE | -0.15 | -0.07 | -0.03 | -0.09 | -0.02 | -0.09 | -0.20^*^ | 0.08 |
|  | LN-NutE | -0.10 | 0.03 | -0.06 | -0.04 | 0.10 | -0.11 | -0.10 | 0.03 |
| V | HN-NUE | 0.04 | -0.08 | 0.13 | 0.11 | 0.08 | 0.21^**^ | 0.06 | 0.12 |
|  | LN-NUE | 0.06 | -0.15^*^ | 0.20^*^ | 0.21^**^ | 0.12 | 0.11 | 0.03 | 0.12 |
|  | HN-NupE | 0.21^**^ | -0.02 | 0.22^**^ | 0.10 | 0.14 | 0.06 | 0.26^**^ | -0.04 |
|  | LN-NupE | 0.14 | -0.12 | 0.21^**^ | 0.24^**^ | 0.11 | 0.13 | 0.18 | -0.04 |
|  | HN-NutE | -0.19^*^ | -0.06 | 0 | 0.01 | -0.13 | 0.04 | -0.24^*^ | 0.15 |
|  | LN-NutE | -0.20^*^ | -0.06 | -0.08 | -0.06 | -0.09 | 0.03 | -0.18 | 0.20^*^ |

Data for NUE-related traits came from Li et al., (2015). * Significant at *P* <0.05, ** Significant at *P* <0.01

**Table S4**. Pearson’s correlation coefficients of RSA-related traits in three culture systems and P efficiency related trait in the field evaluated under normal- phosphorus (NP) and low- phosphorus (LP) levels. Phosphorus use efficiency (PUE), phosphorus uptake efficiency (PupE), phosphorus utilization efficiency (PutE).

| Culture systems | Trait | RDW | PRL | SRL | SRN | CRL | CRN | LRL | LRD_PR_ |
| --- | --- | --- | --- | --- | --- | --- | --- | --- | --- |
| P | NP-PUE | 0.11 | 0.06 | -0.30 | -0.10 | 0.25 | -0.24 | 0 | -0.17 |
|  | LP-PUE | -0.10 | -0.06 | -0.31^*^ | 0.06 | 0.03 | -0.45^**^ | 0.02 | 0.04 |
|  | NP-PupE | 0.02 | -0.10 | 0.21 | 0.19 | 0.04 | -0.03 | 0 | 0.05 |
|  | LP-PupE | 0.02 | 0.02 | 0.11 | -0.07 | -0.05 | -0.03 | 0.08 | -0.06 |
|  | NP-PutE | -0.14 | -0.08 | -0.08 | -0.14 | 0.18 | 0.11 | -0.17 | 0.19 |
|  | LP-PutE | -0.01 | -0.05 | -0.23 | -0.02 | 0.13 | -0.17 | 0.08 | -0.04 |
| H | NP-PUE | -0.12 | 0.02 | 0.13 | 0.11 | 0.05 | -0.07 | -0.07 | 0.03 |
|  | LP-PUE | -0.18 | -0.13 | -0.13 | -0.05 | 0 | -0.05 | -0.19 | 0.13 |
|  | NP-PupE | 0.16 | -0.04 | 0.22^*^ | 0.16 | 0.03 | -0.04 | 0.02 | -0.03 |
|  | LP-PupE | 0.28^**^ | 0.05 | 0.25^*^ | 0.20 | 0.03 | -0.11 | 0.03 | -0.21^*^ |
|  | NP-PutE | -0.14 | -0.06 | -0.03 | -0.11 | 0.10 | 0.06 | -0.06 | 0.07 |
|  | LP-PutE | 0.12 | -0.08 | 0.14 | 0.17 | 0.10 | -0.04 | -0.09 | -0.20^*^ |
| V | NP-PUE | -0.05 | -0.05 | 0.11 | 0.17 | -0.03 | 0.15 | -0.08 | 0.20 |
|  | LP-PUE | -0.17 | -0.10 | -0.07 | -0.02 | -0.15 | 0.04 | -0.14 | 0.13 |
|  | NP-PupE | 0.07 | -0.19 | 0.02 | 0.09 | -0.11 | 0.02 | 0.13 | -0.04 |
|  | LP-PupE | 0.28^**^ | 0.01 | 0.27^**^ | 0.15 | 0.01 | -0.10 | 0.34^**^ | 0.02 |
|  | NP-PutE | -0.06 | -0.10 | 0.01 | -0.01 | -0.02 | 0.07 | -0.05 | 0.13 |
|  | LP-PutE | 0.17^*^ | -0.11 | 0.18^*^ | 0.18^*^ | 0 | 0.04 | 0.09 | 0.01 |

Data for PUE-related traits came from Gu et al., (2016). * Significant at *P* <0.05, ** Significant at *P* <0.01.
